# Prevalence, role impairment, and mental health service utilization of depressive disorders among community-dwelling older adults in China

**DOI:** 10.3389/fpsyt.2026.1897102

**Published:** 2026-09-03

**Authors:** Dandan Wang, Xiaofei Hou, Zhaorui Liu, Yueqin Huang, Shuiyuan Xiao, Lingjiang Li, Tingting Zhang, Jie Yan, Yaqin Yu, Xiufeng Xu, Zhizhong Wang, Yifeng Xu, Tao Li, Xiangdong Xu, Limin Wang, Yongping Yan, Minghui Li, Guangming Xu, Huifang Yin

**Affiliations:** 1 Tianjin Anding Hospital, Mental Health Center of Tianjin Medical University, Tianjin, China; 2 Peking University Sixth Hospital, Peking University Institute of Mental Health, NHC Key Laboratory of Mental Health (Peking University), National Clinical Research Center for Mental Disorders (Peking University Sixth Hospital), Beijing, China; 3 Department of Social Medicine and Health Management, School of Public Health, Central South University, Changsha, China; 4 Mental Health Institute, The Second Xiangya Hospital, Central South University, Changsha, China; 5 School of Government, Peking University, Beijing, China; 6 Department of Epidemiology and Biostatistics, School of Public Health, Jilin University, Changchun, China; 7 Department of Psychiatry, The First Affiliated Hospital of Kunming Medical University, Kunming, China; 8 Department of Epidemiology and Health Statistics, School of Public Health and Management, Ningxia Medical University, Yinchuan, China; 9 Shanghai Mental Health Center, Shanghai Jiao Tong University School of Medicine, Shanghai, China; 10 Mental Health Centre, West China Hospital, Sichuan University, Chengdu, China; 11 The Fourth People’s Hospital in Urumqi, Urumqi, China; 12 National Center for Chronic and Noncommunicable Disease Control and Prevention, Chinese Center for Disease Control and Prevention, Beijing, China; 13 Department of Epidemiology, Ministry of Education Key Laboratory of Hazard Assessment and Control in Special Operational Environment, School of Public Health, Fourth Military Medical University, Xi’an, China

**Keywords:** depressive disorders, epidemiology, health service utilization, older adults, role impairment

## Abstract

**Background:**

Depressive disorders pose a significant public health burden, particularly among older adults. However, their prevalence, associated factors, role impairment, and mental health service utilization patterns among community-dwelling older ay -30dults in China remain understudied.

**Methods:**

The data for this study were obtained from the China Mental Health Survey (CMHS) among participants aged 60 years and older. The World Health Organization Composite International Diagnostic Interview Version 3.0 (CIDI 3.0) was administered to generate mental disorder diagnoses consistent with the Diagnostic and Statistical Manual of Mental Disorders, Fourth Edition, Text Revision (DSM-IV-TR). We calculated weighted prevalence of depressive disorders and applied multivariable logistic regression to identify associated factors, with further analyses of role impairment and mental health service utilization.

**Results:**

The final sample encompassed 7,454 older adults. The weighted lifetime and 12-month prevalence of depressive disorders among Chinese community-dwelling older adults were 7.9% (95% CI: 6.4%-9.5%) and 4.1% (95% CI: 3.2%-4.9%), respectively. Multivariable logistic regression analysis showed that older adults with three or more physical illnesses exhibited greater odds of past-year depressive disorders (OR = 11.4, 95% CI: 7.0-18.6). Elevated odds were also observed among those with comorbid anxiety and substance use disorders (OR = 8.4, 95% CI: 5.5-12.8; OR = 8.9, 95% CI: 3.9-20.0, respectively). Among respondents with 12-month depressive disorders, 67.9% had role impairment in household duties, 73.2% in work, 62.2% in relationships, and 60.7% in social life. Only 11.3% of those diagnosed with depressive disorders in the past 12 months utilized any form of mental health service.

**Conclusions:**

Depressive disorders are prevalent among community-dwelling Chinese older adults and are associated with various factors. Patients commonly experienced multi-domain role impairment, whereas mental health service uptake was low, calling for strengthened screening, intervention and accessible care for older adults.

## 1 Introduction

The global population is aging rapidly, presenting various public health challenges, among which depressive disorders in older adults are particularly prominent. Depressive disorders not only severely affect older adults’ quality of life but also burden society with high healthcare costs and productivity losses (1–3). According to the 2019 Global Burden of Disease (GBD) estimates, depressive disorders ranked 13th among all causes of disability-adjusted life years (DALYs) and second in terms of years lived with disability (YLDs) globally across all ages (4). Among mental disorders, they contributed the most to YLD burden in older adults (4).

According to the Diagnostic and Statistical Manual of Mental Disorders, Fourth Edition, Text Revision (DSM-IV-TR) diagnostic criteria, depressive disorders fall under the umbrella of mood disorders, covering three primary clinical subtypes: major depressive disorder, dysthymic disorder, and depressive disorder not otherwise specified (5). Despite the high global disease burden, large-scale epidemiological studies adopting standardized diagnostic criteria for older adults remain limited. Furthermore, diagnostic-based investigations mostly focus on major depressive disorder, while the prevalence of the other two depressive subtypes among older populations is rarely documented (6). Restricted by field survey conditions, most existing population studies rely on operational depression definitions based on scale cut-off scores, which may lead to heterogeneous prevalence estimates.

With regard to epidemiological data, a systematic review and meta-analysis, integrating data from 48 studies, revealed that the global prevalence of depression in individuals aged 60 and above reached 28.4% (7). In China, a previous study demonstrated that the prevalence of depression among older adults aged 60 years and above exhibits substantial variation across diverse cultural and regional settings, ranging from 6% to 27% (8). By contrast, meta-analyses based on formal diagnostic interviews have reported the 12-month prevalence of major depressive disorder among Chinese older adults to be 2.3% (9). The considerable between-study variation and substantial disease burden underscore an urgent need for rigorous population-based diagnostic research to characterize depressive disorders among community-dwelling older adults in China.

Numerous associated factors contribute to the development of depressive disorders in older adults. Demographic factors assume a pivotal role. Older women, in particular, are more predisposed due to hormonal changes during menopause, as well as social pressures and gender-related expectations (10, 11). Older individuals who are single, widowed, or divorced are at an elevated risk, primarily due to the lack of the emotional and social support typically provided by a spouse (10, 12). The level of educational attainment is also of significant importance. Lower-educated older adults have greater odds of depression, mediated by socioeconomic standing, healthcare access and social support (13).

Beyond demographic factors, multiple clinical and psychosocial correlates have been documented. Sleep disturbances, anxiety disorders and substance use disorders commonly co-occur with late-life depression, complicating clinical management and potentially exacerbating illness severity (14–16). Poor self-perceived health, adverse childhood experiences, and negative life events are also identified as risk factors (17, 18), while regular engagement in physical activity can be protective (19, 20). Comprehensive understanding of the risk and protective factors is indispensable for the formulation of evidence-based preventive and intervention strategies.

Functional impairment in daily activities, such as self-care and social engagement, is a hallmark of late-life depression and a critical determinant of patients’ quality of life (21). A previous study suggests that depression may contribute to age-related declines in physical and cognitive functioning, leading to increased dependency, reduced social participation, and higher caregiver burden (22). In Chinese society, older adults hold vital roles like family caregivers, preservers of traditional values, and sometimes remain in the workforce. When suffering from depressive disorders, fulfilling these roles becomes challenging. However, while the association between depressive symptoms and functional disability among Chinese older adults has been documented (23), reports on the functional status of older adults meeting clinical diagnostic criteria for depressive disorders remain scarce in China. This gap limits policymakers’ ability to prioritize resource allocation for mental health interventions tailored to older populations.

Mental health services utilization constitutes a critical public health issue. Data from the World Mental Health Survey (WMHS) across 17 countries indicate that 12-month mental health service use among community adults ranges from 1.6% to 17.9%, with lower rates in less-developed countries (24). Service-seeking behaviour is shaped by subjective factors (self-stigma, mental health literacy), structural barriers (limited facilities, workforce constraints and poor accessibility), and demographic factors including age, income and mental disorder status (25).

In the context of China, the China Mental Health Survey (CMHS) indicated that merely 9.5% of adults with depressive disorders received healthcare treatment within the past 12 months (11). The mental health service needs of older adults remain largely unmet (26). Chinese older adults encounter multiple interrelated barriers to formal mental health services (27). Stigma discourages help-seeking, and psychological distress tends to present somatically rather than as mood symptoms (28). Distinct local barriers contribute to the low access to professional services among older adults in the community. Further challenges include sparsely and unevenly distributed geriatric psychiatric resources between urban and rural settings, and limited capacity of primary care providers to screen for depressive disorders (11, 25).

Research specifically targeting depressive disorders within the Chinese community-dwelling older adults remains conspicuously scant. Most prior studies focus on older adults during public health emergencies or in specific subgroups like those with chronic illnesses, or empty-nesters (29, 30). Thus, accurately determining the prevalence of these disorders in older adults is crucial. Using data from the CMHS, this study aims to describe the prevalence of depressive disorders in Chinese community-dwelling older adults, analyze related factors, and explore mental health service utilization. Given the global unmet need for older adults’ mental health services and the situation in China, this research is expected to contribute to better prevention and treatment strategies, improving older adults’ mental health and quality of life.

## 2 Methods

### 2.1 Study design and samples

The CMHS was designed with the objective of obtaining a nationally representative sample of Chinese nationals 18 years of age and older, who had lived in community households for at least six months. Conducted from July 22, 2013, to March 5, 2015, this cross-sectional representative household survey encompassed 31 provinces in mainland China. Individuals who were institutionalized or unable to undergo the interview were excluded from the study.

The China Mental Health Survey (CMHS) employed a multi-stage disproportionate stratified sampling method within the framework of the Disease Surveillance Point (DSP) system. This approach involved the selection of 31 provinces, autonomous regions, and municipalities to ensure a nationally representative sample of the Chinese population. Ultimately, CMHS identified a total of 40,964 households across 1,256 communities or villages in 628 streets or towns throughout 157 locations nationwide. After excluding ineligible households, 38,593 valid households remained. From these eligible households, 32,552 individuals selected through computer-assisted sampling for CIDI assessment. Subsequent exclusion of participants who did not complete the CIDI interview yielded a final sample of 28,140 individuals who completed full diagnostic assessments. Full study profile of the sample can be found in our prior publications (31, 32). For the specific purposes of this current study, we excluded 20,686 participants younger than 60 years from this overall CIDI sample. Those aged 60 years and older were retained, yielding a final analytic sample of 7,454 individuals. The data analysis for this study was conducted between October 2024 and February 2025.

Participants in the CMHS were selected through a multi-stage clustered area probability sample of households based on the resident registry. The studies involving humans were approved by the Ethics Committee of the Sixth Hospital of Peking University in Beijing. The studies were conducted in accordance with the local legislation and institutional requirements. Written informed consent to participate in this study was provided by the participants. The research protocol was approved by the Ethics Committee of the Sixth Hospital of Peking University in Beijing, China (IMH-IRB-2013-13-1). The study design of the CMHS has been described in detail in previous literature (31, 33).

### 2.2 Procedures and assessments

Trained interviewers conducted face-to-face interviews with the participants who consented to participate in the study. The Chinese computer-assisted personal interview (CAPI) version of the Composite International Diagnostic Interview (CIDI) 3.0, a fully structured diagnostic tool (34), was employed for data collection. The CIDI assessment was divided into two parts. All participants completed Part I, which screened for most mental disorders. Part II targeted a subsample comprising participants with lifetime disorders diagnosed in Part I and a 25% random sample of disorder-free respondents, collecting detailed sociodemographic, correlate, and pharmacoepidemiological data (11, 31–33).

### 2.3 Measurement

#### 2.3.1 Diagnostic interview

The diagnostic information regarding depressive disorders was sourced from Part I of the CIDI. Through structured interviews, respondents were evaluated to ascertain whether they fulfilled the DSM-IV-TR criteria for depressive disorders, either over their lifetime or within the 12-month period preceding the interview.

Depressive disorders were diagnosed via CIDI 3.0 based on DSM-IV-TR criteria, encompassing three non-mutually exclusive subtypes: major depressive disorder, dysthymic disorder, and depressive disorder not otherwise specified. Multiple concurrent depressive subtype diagnoses were allowed. Moreover, the comorbidity status of other common mental disorders, including anxiety disorders and substance use disorders, was also diagnosed using the CIDI.

#### 2.3.2 Sociodemographic and health characteristics

Sociodemographic covariates and chronic physical conditions were obtained from Part I of CIDI 3.0 interview and available for all 7,454 participants.

Sociodemographic characteristics included age, gender, region, urbanicity, education level, marital status, and household income. Age was stratified into three groups: 60–64 years, 65–69 years, and 70 years and above. Region was considered by categorizing participants as residents of the eastern, central, or western regions based on the origin of the study data. Urbanicity was determined by classifying participants as rural or urban residents according to their addresses at the time of data collection. Education level was classified into four categories: illiterate/below primary school, completed primary school, completed junior high school, and senior high school and above. Marital status was dichotomized into two groups: married or cohabitating, and unmarried, which encompasses those who have never married, as well as those who are separated, divorced, or widowed. Household income was stratified into three distinct levels based on the annual income percentiles of the total sample in the CMHS: low income, defined as less than 10,500 Yuan; middle income, ranging from 10,500 to 42,500 Yuan; and high income, exceeding 42,500 Yuan.

Chronic physical illnesses were assessed using a standard checklist covering conditions including heart disease, chronic lung disease, high blood pressure, tuberculosis, diabetes, asthma, stroke, rheumatic fever or arthritis, stomach ulcers or intestinal ulcers, and chronic headaches. Participants were then categorized into three groups based on the number of reported illnesses: none, 1-2, or three or more.

#### 2.3.3 Role impairment

Analyses of role impairment and mental health service utilization were limited to participants diagnosed with 12-month depressive disorders via CIDI 3.0 Part I. Role impairment attributable to depression was measured by the Sheehan Disability Scale (SDS) in the CIDI 3.0 Part I depression module across four domains: household duties, work, relationships, and social life (35) to evaluate the degree to which depression induced role impairment concerning household duties, work, relationships, and social life during the worst month of the past year. The four items of the SDS were rated from 0 to 10. Specifically, impairment scores of 0 were designated as none, 1–3 as mild, 4–6 as moderate, 7–9 as severe, and 10 as very severe.

#### 2.3.4 Mental health service utilization

Data on service utilization mainly came from CIDI 3.0 Part I depression module. Supplementary medication information was extracted from the CIDI Part II pharmacoepidemiology module only to complement missing records. Based on previous research, mental health service sectors were categorized into four groups: specialty mental health (treatments provided by any mental health professional including psychiatrists, psychologists, social workers or counselors in a specialist setting), general medical (treatments provided by any health professional including medical specialists or nurses in a human services setting), human services (treatment provided by religious personnel, social workers or counselors in any setting excluding mental health and human services), and complementary and alternative medicine (CAM, any other style, online, or self-help group). Moreover, health care treatment is defined as making at least one visit for mental disorder treatment in the past 12 months in either the specialty mental health sector or the general medical sector or using psychotropic medications during the same time frame (11). However, owing to the relatively small sample sizes within the human services and CAM groups, these two categories were merged for the purposes of statistical analysis.

### 2.4 Statistical analyses

All statistical analyses were weighted. Final analytical weights were constructed by combining three sequential components: sampling design weights, non-response adjustment weights, and post-stratification weights, with weight trimming performed to mitigate extreme values (31). Sampling design weights were calculated as the inverse probability of selection within the seven-stage clustered CMHS sampling scheme, derived from the product of weights at each sampling stage. Composite non-response weights were generated by multiplying village-, household-, and individual-level adjustment weights. Post-stratification weights were estimated based on the age-sex-residence distribution data from China’s 2010 census population survey (16). All analyses were restricted to participants who completed the CIDI Part I interview. Given that core diagnostic outcomes and primary covariates were derived from Part I data, Part II subsample weights were not incorporated into any analyses in the present study.

The prevalence of depressive disorders and their subtypes, as well as the mental health service utilization among older adults with depressive disorders was described. The 95% confidence intervals (CIs) of the prevalence estimates were computed using the Taylor series linearization method, which accounted for both sampling weights and sample clustering. Univariable and multivariable logistic regression analyses based on complex sampling were performed to explore the associations between sociodemographic and clinical factors and the presence of 12-month depressive disorders. Univariable logistic regression models were constructed by incorporating one variable at a time to generate crude odds ratios (ORs). All sociodemographic and health-related variables were simultaneously included in the multivariable model. A *p*-value of less than 0.05 was defined as the threshold for statistical significance. Collinearity diagnostics for the weighted logistic regression models were conducted to assess multicollinearity. Relevant indicators, including tolerance values, variance inflation factor (VIF), and correlation matrix eigenvalues are available. Sensitivity analyses were further performed to test the robustness of core findings, using a simplified multivariable model that excluded the variables with univariate *p*-value>0.1. All statistical analyses were executed using SAS 9.4.

## 3 Results

### 3.1 Sample characteristics

**Figure 1:**
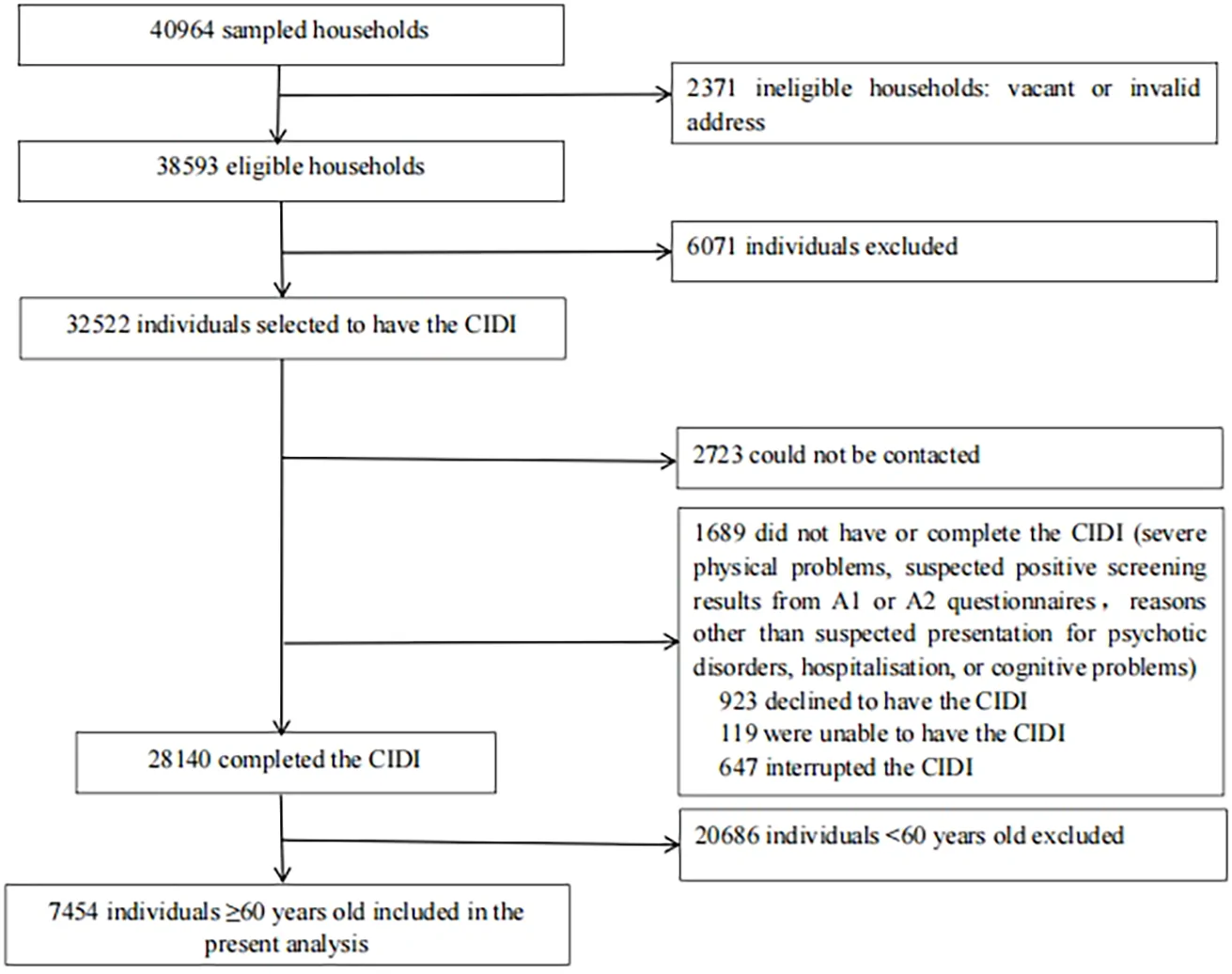
Participant flow diagram. Flowchart redrawn by the authors based on the sampling framework of the China Mental Health Survey (CMHS) reported in Liu et al. (31). Suspected positive screening results from A1 or A2 questionnaires.

The final sample encompassed 7,454 older adults who completed the CIDI 3.0 interview, and all the associated data were subjected to analysis. The participant flow diagram is presented in **Figure 1**. Females accounted for 50.5% of the sample. The proportion of those aged 60–64 years was 40.6%, while 26.7% were in the 65-69-year-old age group. Moreover, 52.9% of the sample resided in rural areas, and 78.9% were married or cohabiting. **Supplementary Table e.1** presents additional background details of the sample.

### 3.2 Prevalence of depressive disorders among Chinese older adults

As shown in **Table 1**, within community-dwelling older adults in China, the weighted lifetime prevalence and 12-month prevalence of depressive disorders were 7.9% (95% CI: 6.4%-9.5%) and 4.1% (95% CI: 3.2%-4.9%), respectively. Major depressive disorder emerged as the most prevalent depressive disorder, with a weighted lifetime prevalence of 5.5% (95% CI: 4.5%-6.6%) and a weighted 12-month prevalence of 3.4% (95% CI: 2.5%-4.2%). The detailed prevalence data for any 12-month depressive disorder by different sociodemographic characteristics are presented in **Table 2**.

**Table 1:** Prevalence of depressive disorders among older adults in China.

Depressive disorder category	Lifetime		12-month prevalence	
	Frequency n	Weighted prevalence % (95% CI)	Frequency n	Weighted prevalence % (95% CI)
Depressive disorders ^a^	568	7.9 (6.4-9.5)	293	4.1 (3.2-4.9)
Major depressive disorder	394	5.5 (4.5-6.6)	237	3.4 (2.5-4.2)
Dysthymic disorder	142	2.1 (1.5-2.7)	106	1.6 (1.1-2.2)
Depressive disorder NOS	155	2.1 (1.1-3.2)	48	0.6 (0.3-1.0)

NOS, Not otherwise specified; CI, Confidence Interval. ^a^The cumulative number of the three subgroups is not the total number of cases of depressive disorders because of some instances of comorbidity of major depressive disorder with dysthymic disorder.

**Table 2:** Prevalence of any 12-month depressive disorder by sociodemographic characteristics (N = 7454).

Sociodemographic characteristics	Frequency n	Weighted prevalence % (95% CI)
Age		
60–64 years	135	4.7 (3.5-5.8)
65–69 years	85	4.3 (3.0-5.5)
70 years and above	73	3.5 (2.4-4.7)
Gender		
Male	110	3.2 (2.3-4.1)
Female	183	4.9 (3.5-6.4)
Region		
Eastern	80	3.8 (2.5-5.0)
Central	113	4.0 (2.4-5.6)
Western	100	4.6 (3.0-6.1)
Urbanicity		
Rural	173	4.6 (3.5-5.8)
Urban	120	3.4 (2.3-4.4)
Education level		
Senior high school and above	16	3.1 (0.1-6.1)
Junior high school	40	4.0 (2.0-6.0)
Primary school	87	5.1 (3.9-6.3)
Illiterate/below primary school	150	3.8 (2.7-4.9)
Marital status		
Married or cohabitating	199	3.8 (2.8-4.7)
Others ^a^	94	5.5 (3.6-7.3)
Household income ^b^		
Low	161	5.1 (3.8-6.3)
Middle	85	3.4 (2.0-4.8)
High	47	2.7 (1.3-4.2)

CI, Confidence Interval; ^a^Others included never married, separated, widowed, and divorced; ^b^The household income was categorized into three levels based on yearly income percentiles: low (<10,500 yuan), middle (10,500-42,500 yuan), and high (42,500 yuan and above).

### 3.3 Factors associated with depressive disorders among Chinese older adults

As shown in **Table 3**, multiple logistic regression analyses revealed that, compared with older adults without physical illnesses, those with three or more physical illnesses had significantly greater odds of past-year depressive disorders (OR = 11.4, 95% CI: 7.0-18.6), while elevated odds were also observed among those with one or two physical illnesses (OR = 2.8, 95% CI: 1.7-4.5). Older adults with comorbid anxiety disorders or substance use disorders exhibited significantly greater odds of experiencing a depressive episode within the past year (OR = 8.4, 95% CI: 5.5-12.8; OR = 8.9, 95% CI: 3.9-20.0, respectively).

**Table 3:** Sociodemographic and health correlates with 12-month depressive disorder in Chinese older adults (N = 7454)^a^.

Variables	Univariate logistic ^b^		Multivariable logistic ^c^	
	OR (95% CI)	*P*	OR (95% CI)	*P*
Age				
60–64 years	Reference		Reference	
65–69 years	0.9 (0.6-1.3)	0.601	0.9 (0.6-1.3)	0.552
70 years and above	0.8 (0.5-1.1)	0.112	0.8 (0.5-1.1)	0.171
Gender				
Male	Reference		Reference	
Female	1.6 (1.1-2.4)	**0.026**	1.4 (0.9-2.3)	0.165
Region				
Eastern	Reference		Reference	
Central	1.1 (0.6-1.8)	0.829	1.1 (0.6-2.0)	0.689
Western	1.2 (0.7-2.0)	0.434	1.3 (0.8-1.9)	0.284
Urbanicity				
Rural	Reference		Reference	
Urban	0.7 (0.5-1.1)	0.086	0.8 (0.5-1.2)	0.324
Education level				
Senior high school and above	Reference		Reference	
Junior high school	1.3 (0.4-4.1)	0.649	1.2 (0.3-4.3)	0.819
Primary school	1.7 (0.6-4.8)	0.326	1.6 (0.5-5.3)	0.456
Illiterate/below primary school	1.2 (0.4-3.5)	0.697	0.9 (0.3-3.3)	0.907
Marital status				
Married or cohabitating	Reference		Reference	
Others ^d^	1.5 (1.0-2.3)	0.077	1.4 (0.8-2.2)	0.242
Household income ^e^				
Low	Reference		Reference	
Middle	0.7 (0.4-1.1)	0.086	0.7 (0.4-1.1)	0.156
High	0.5 (0.3-1.0)	**0.044**	0.5(0.3-1.0)	0.060
Number of chronic physical diseases				
None	Reference		Reference	
1-2	3.1 (1.9-5.0)	**<0.001**	2.8 (1.7-4.5)	**<0.001**
3 and more	14.2 (8.9-22.7)	**<0.001**	11.4 (7.0-18.6)	**<0.001**
12-month anxiety disorders				
No	Reference		Reference	
Yes	12.2 (8.0-18.5)	**<0.001**	8.4 (5.5-12.8)	**<0.001**
12-month substance use disorders				
No	Reference		Reference	
Yes	9.8 (4.1-23.6)	**<0.001**	8.9 (3.9-20.0)	**<0.001**

OR, Odds Ratio; CI, Confidence Interval; ^a^These analyses were based on part one weighted data; ^b^Univariable logistic regression models were unadjusted models, constructed by incorporating one listed variable at a time to generate crude ORs; ^c^All listed variables (age group, gender, region, urbanicity, education level, marital status, household income, number of chronic physical diseases, presence of 12-month anxiety disorders, and presence of 12-month substance use disorders) were simultaneously entered into the multivariable logistic regression model to obtain adjusted ORs; ^d^Others included never married, separated, widowed, and divorced; ^e^The household income was categorized into three levels based on yearly income percentiles: low (<10,500 yuan), middle (10,500-42,500 yuan), and high (42,500 yuan and above). Bold values indicate statistical significance (P < 0.05).

Collinearity diagnostics were performed for the multivariable logistic regression models based on weighted analyses. Tolerance values, VIF (Table e.2), and eigenvalues of the correlation matrix (Table e.3) are provided in the **Supplementary Materials**. The diagnostic results indicate a low risk of multicollinearity.

Sensitivity analyses were performed to verify the robustness of the main findings. We refitted a simplified multivariable logistic regression model by omitting three variables that yielded univariate P>0.1 (age, region, and education level). The results are presented in **Supplementary Table e.4**. Association patterns largely remained consistent between the fully adjusted model and sensitivity model. The sole difference related to household income, with its P value changing from 0.060 in the full model to 0.047 in the simplified model. In the simplified model, higher household income was linked to lower odds of 12-month depressive disorders (OR = 0.5, 95% CI: 0.3-1.0) compared with older adults with low household income.

### 3.4 Role impairment of 12-month depressive disorders

**Figure 2:**
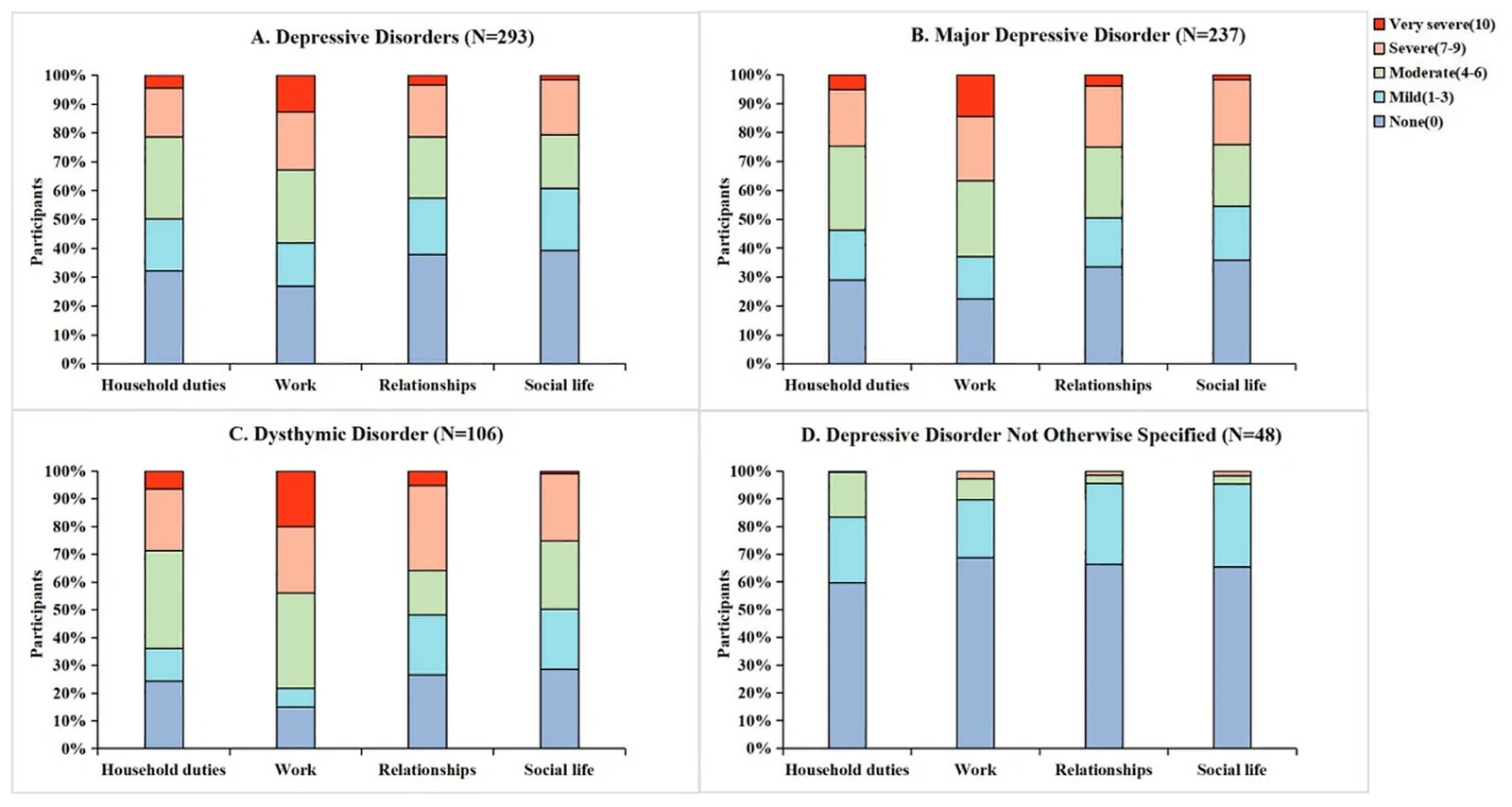
Role impairment of 12-month depressive disorders measured by SDS. SDS, Sheehan Disability Scale; These estimates were based on Part I weighted data.

As depicted in **Figure 2**, among respondents with 12-month depressive disorders, 67.9% experienced role impairment in the household duties domain, 73.2% in the work domain, 62.2% in the relationships domain, and 60.7% in the social life domain. Major depressive disorder and dysthymic disorder exhibited similar proportions of severe, moderate, and mild impairments across the four domains of SDS. The proportions of both severe and moderate impairments in these two disorders were higher than those in depressive disorder not otherwise specified. The detailed data on the severity levels of role impairment for each type of depressive disorder are presented in **Supplementary Tables e.5–e.8**.

### 3.5 Treatment for depressive disorders within the past 12 months

Among the participants diagnosed with depressive disorders over the past 12 months, a mere 11.3% had availed themselves of any form of mental health service. More precisely, 3.3% of the participants accessed specialty mental health services, 5.5% utilized general medical services, and 1.3% made use of human services or CAM (**Table 4**).

**Table 4:** Mental health service utilization in the past 12 months of 12-month depressive disorders (n=293)^a^.

Sector of mental health service	n	%	95% CI
Health care ^b^	42	11.1	6.4-15.8
Mental health specialty	11	3.3	0.1-6.5
General medical	26	5.5	2.6-8.3
Human service and CAM	2	1.3	0.0-3.5
**Any**	43	11.3	6.6-16.0

CI, Confidence Interval; CAM, complementary and alternative medicine; ^a^These estimates were based on weighted data; ^b^Health care was defined as making at least one visit for mental disorder treatment in the past 12 months in either the specialty mental health sector or the general medical sector, or using psychotropic medications during the same period. Bold text indicates the overall proportion of participants who utilized any mental health service.

## 4 Discussion

Employing a nationally representative sample from CMHS, this study offers a multidimensional assessment of depressive disorders among community-dwelling Chinese adults aged 60 years and older, encompassing population-level prevalence, key sociodemographic and clinical correlates, role impairment across multiple domains, and patterns of mental health service utilization. Earlier CMHS analyses have documented mental disorders, as well as depressive disorders specifically, in the general adult population (11, 32), yet few comprehensively examined depressive disorders among older adults. Although one secondary analysis of CMHS data focused on an older subgroup, it was limited to older adults living with non-communicable diseases (36). Against this backdrop, the present study fills this research gap, and its findings highlight important public health challenges and opportunities for targeted intervention.

Our study documents that 7.9% of Chinese community-dwelling older adults experienced lifetime depressive disorders, with 4.1% reporting episodes in the past 12 months. While these estimates align with prior regional epidemiological studies from China (27), our nationally representative data provide more robust evidence. Our findings demonstrate a higher prevalence of depressive disorders in older adults (60 years and above) compared to the general adult population (18 years and above: lifetime 6.8%, 12-month 3.6%) from the same CMHS dataset reported previously (11, 32). These results highlight the growing mental health burden associated with population aging and underscore the imperative for targeted geriatric mental health interventions in China’s public health framework. The observed prevalence remains markedly lower than in Western populations (37, 38). First, this pronounced cross-national discrepancy likely arises from a confluence of methodological and sociocultural determinants. Culturally mediated variations in symptom manifestation and illness attribution patterns may contribute to substantial under-detection in the Chinese population, where pervasive mental health stigma persists (28). Second, subtle inconsistencies in the implementation of practical diagnostic thresholds may exist across research teams, even when adhering to identical DSM-IV criteria. This variability could potentially alter the final case identification rates. Third, there is substantial heterogeneity in survey methodologies, encompassing sampling designs and interview procedures. These variations create inherent gaps in cross-national prevalence estimates.

Identifying factors related to depressive disorders is crucial for understanding the etiology and potential prevention strategies. Our study reveals a notable comorbidity between geriatric depressive disorders and chronic physical illnesses, with a dose-dependent relationship. Older adults with three or more chronic physical illnesses exhibited significantly greater odds of depressive disorders, whereas those with one or two also showed elevated odds, consistent with prior research (10, 30). Evidence indicates that the relationship between chronic physical illness and depressive disorders is bidirectional. From a pathophysiological perspective, chronic physical ailments act as significant associated factors for depressive disorders, may be linked to symptoms through pathways like physical disability limiting function, quality-of-life decline causing distress, and persistent pain providing negative stimuli (39, 40). Conversely, depressive disorders may be linked to chronic physical conditions. Manifestations such as limited social engagement, sleep disturbance, and unhealthy behaviors impede functional recovery and health maintenance (41, 42). These findings suggest that integrated approaches to manage both physical and mental health problems are necessary in clinical practice.

This study reveals a high prevalence of comorbidity between depressive disorders and other psychological conditions among older adults. Multiple interrelated biological factors contribute to this phenomenon. Notably, approximately 65% of older adults with depressive disorders also present with anxiety disorders (43), which can be attributed to neurobiological changes, inflammatory processes, and alterations in the stress response system (43–45). Chronic inflammation, in particular, is not only associated with the development of depressive disorders but also implicated in the pathogenesis of anxiety disorders. The relationship between depressive and anxiety disorders in older adults is bidirectional. Individuals with depressive disorders often experience health-related anxiety, which, in turn, may contribute to depressive symptoms. This feedback loop increases the likelihood of co-morbidity, as the presence of one disorder elevates the risk of developing the other.

Our findings also align with previous research, demonstrating the frequent co-occurrence of substance abuse with depressive disorders, highlighting the necessity of addressing substance abuse in the treatment of depressive disorders (6, 16). The interaction between depressive disorders and substance abuse can create a vicious cycle. Older adults with depressive disorders may resort to alcohol or drugs as a maladaptive coping mechanism to alleviate emotional distress, leading to increased substance dependence. Conversely, substance abuse further intensifies depressive symptoms, complicating treatment and recovery efforts (46, 47). This cycle poses a significant threat to the well-being of older adults, who may already be burdened by multiple health issues and the effects of aging. Therefore, it is crucial to tackle these comorbidities through an effective healthcare system that aims to mitigate the risk of depressive disorders among older adults.

The association between household income and late-life depressive disorders varied across model specifications. While household income showed marginal significance in the fully adjusted model, it became statistically significant in the simplified sensitivity model excluding age, region and education level. This change demonstrates that the income effect is partially confounded by these demographic factors. Lower household income may increase depression risk via financial strain and restricted access to health and social resources (48), yet such association overlaps with disparities related to age, residence and education (49). Given this marginal and model-dependent finding, we urge cautious interpretation, and suggest that financial interventions should be integrated with multi-dimensional support strategies for vulnerable older populations.

The widespread role impairment across multiple domains demonstrates the substantial disability burden of depressive disorders. Particularly concerning is the high proportion reporting work-related difficulties, suggesting significant economic impacts through reduced productivity. Older adults suffering from depressive disorders typically experience decreased energy, resulting in diminished productivity and increased absenteeism, which further impacts their job performance (50). From a social perspective, the reduced ability of older adults to participate in and maintain social networks, along with the loss of meaningful intimate relationships, may be associated with chronic late-life depressive disorders that can undermine relationships and foster isolation (51). Within the familial context, loneliness intensifies due to a lack of connection to family and community, compounded by reduced physical mobility. The association between loneliness and depressive disorders is particularly significant for health in later life, as these factors synergistically diminish well-being among the older adults (52, 53). Furthermore, research indicates that older adults with depressive disorders coexisting with cognitive impairment or physical illness, may encounter greater challenges in sustaining their roles at home and in the workplace (54). Notably, our findings reveal that both major depressive disorder and dysthymic disorder inflict severe role impairment. In community-dwelling older adults, dysthymia and subthreshold depressive disorders are more prevalent than major depressive disorder (55). Although dysthymic disorder typically presents with milder symptoms than major depressive episodes, its chronic nature accumulates significant role impairment over time. Our research on Chinese older adults corroborates previous findings (56), demonstrating that the chronic nature of dysthymic disorder may be associated with significant and enduring role impairment. However, evidence regarding the impact of specific treatments on long-term functioning in older adults with dysthymic disorder remains scarce (55).

Our study reveals that only 11.3% of older adults with depressive disorders utilized mental health services, a rate notably lower than that reported in Western countries (57). Multiple intersecting factors may contribute to this situation. Structural barriers significantly impede service utilization. China faces a critical shortage of mental health professionals, with fewer than 2 psychiatrists per 100,000 people, substantially below the global average (10). Compounding this issue is the uneven distribution of health resources, with rural and underdeveloped regions having disproportionately limited access to mental health services (10). Cultural and psychological factors also play a pivotal role. Traditional cultural norms and values prevalent in Chinese society often stigmatize mental illness (28), fostering feelings of shame and inhibiting help-seeking behavior. Older adults, in particular, may be more influenced by these cultural attitudes, leading them to downplay or deny their mental health symptoms. These factors collectively hinder their capacity to identify symptoms of psychological distress, thereby exacerbating the already low rates of mental health service utilization. In 2017, the Chinese government issued the 13th Five-Year Plan for Healthy Ageing, which underlined that healthy aging had become a key priority on the country’s political agenda (58). Despite the advancements brought about by healthcare reform, which have led to increased utilization of general health services (59), mental health service use among Chinese older adults still lags significantly behind that in numerous developed countries. Given this gap, more attention to their mental health services is urgently needed.

## 5 Limitations

This study is subject to several limitations. Firstly, lifetime and 12-month depressive disorder diagnoses relied on retrospective self-report, so recall bias constitutes an inevitable limitation common to all interview-based surveys. Participants’ prevailing mood at the time of face-to-face interviews may also distort their recall of prior depressive symptoms and reduce reporting accuracy. Secondly, the sample size constraints precluded a comprehensive analysis of factors associated with role impairment and mental health services. This limitation hinders the identification of nuanced relationships and may lead to incomplete understanding of the influencing factors. Thirdly, the absence of data on stigma and attitudes toward treatment in the CMHS restricts in-depth analysis. Such information is crucial for understanding the complex social and psychological factors that influence help-seeking behavior and treatment outcomes. Finally, this study relied on 2013–2015 cross-sectional CMHS data, limiting the extrapolation of results to current conditions, especially mental health service utilization. Over the subsequent decade, China has carried out major healthcare and mental health policy reforms alongside demographic changes, reshaping depression awareness, help-seeking behaviours and access to care. Therefore, the estimates only represent the 2013–2015 service environment rather than the current situation. Notably, despite the temporal lag, these data offer an important historical benchmark for future epidemiological comparisons. Evolving socioeconomic and demographic trends may modify the epidemiology of depressive disorders.

## 6 Conclusions

In a sample of community-dwelling older adults in China between 2013 and 2015, depressive disorders were prevalent. Chronic physical illnesses, comorbid anxiety, and substance use disorders were associated with higher odds of 12-month depressive disorder among older adults. Those with depressive disorders commonly experienced role impairment across multiple life domains. However, mental health service utilization remained low, underscoring the need for enhanced screening, intervention, and access to care for this population.

## Data Availability

The original contributions presented in the study are included in the article/**Supplementary Material**, further inquiries can be directed to the corresponding author/s.
