# Supplementary material for: Prevalence, role impairment, and mental health service utilization of depressive disorders among community-dwelling older adults in China
Source: Front Psychiatry. 2026 Sep 3;17:1897102. doi: 10.3389/fpsyt.2026.1897102 (PMC13582425; doi:10.3389/fpsyt.2026.1897102)
Supplement: Supplementary file 1 [file DataSheet1.pdf]

Table e.1 Sociodemographic characteristics of the sample (N=7454)

Characteristics	Frequency n	Unweighted proportion (%)	Weighted proportion (%)
**Age**			
60-64 years	3030	40.6	34.7
65-69 years	1992	26.7	21.7
70 years and above	2432	32.6	43.7
**Gender**			
Female	3764	50.5	50.9
Male	3690	49.5	49.1
**Region**			
Eastern	2580	34.6	37.3
Central	2649	35.5	34.3
Western	2225	29.8	28.4
**Urbanicity**			
Rural	3940	52.9	56.0
Urban	3514	47.1	44.0
**Education level** [a]			
Senior high school and above	689	9.2	6.2
Junior high school	1258	16.9	13.8
Primary school	1797	24.1	24.1
Illiterate/below primary school	3699	49.6	55.9
**Marital Status**			
Married or cohabitating	5884	78.9	81.6
Others [b]	1570	21.1	18.4
**Household income** [c]			
Low	3326	44.6	47.8
Middle	2286	30.7	32.7
High	1842	24.7	19.5

a: Data missing from 11 respondents; b: Others included never married, separated, widowed, and divorced; c: The household income was categorized into three levels based on yearly income percentiles: low (<10,500 yuan), middle (10,500-42,500 yuan), and high (42,500 yuan and above).

Table e.2 Collinearity diagnostics for multivariable logistic regression for 12-month depressive disorders——Tolerance and VIF

Variable	Tolerance	VIF
Age	0.93138	1.07367
Gender	0.88868	1.12527
Region	0.97023	1.03068
Urbanicity	0.94309	1.06034
Education level	0.86373	1.15777
Marital status	0.89808	1.11348
Household income	0.90075	1.11019
Number of chronic physical diseases	0.96796	1.03310
12-month anxiety disorders	0.96940	1.03157
12-month substance use disorders	0.98943	1.01069

VIF=Variance Inflation Factor

Table e.3 Collinearity diagnostics for multivariable logistic regression for 12-month depressive disorders——Eigenvalues of the correlation matrix

Number	Eigenvalue	Difference	Proportion	Cumulative
1	1.68674295	0.46839876	0.1687	0.1687
2	1.21834419	0.04221904	0.1218	0.2905
3	1.17612515	0.13415674	0.1176	0.4081
4	1.04196841	0.05957337	0.1042	0.5123
5	0.98239504	0.08498583	0.0982	0.6106
6	0.89740922	0.04938200	0.0897	0.7003
7	0.84802722	0.07933840	0.0848	0.7851
8	0.76868882	0.03267661	0.0769	0.8620
9	0.73601221	0.09172541	0.0736	0.9356
10	0.64428679	-	0.0644	1.0000

Table e.4 Sensitivity analyses for multivariable logistic regression: sociodemographic and health correlates with 12-month depressive disorder in Chinese elderly (N=7454) [a]

**Variables**	**OR (95% CI)**	***P***
**Gender**		
Male	Reference	
Female	1.3 (0.8-2.0)	0.352
**Urbanicity**		
Rural	Reference	
Urban	0.8 (0.5-1.2)	0.280
**Marital status**		
Married or cohabitating	Reference	
Others [b]	1.2 (0.8-2.0)	0.395
**Household income** [c]		
Low	Reference	
Middle	0.7 (0.4-1.2)	0.164
High	0.5 (0.3-1.0)	**0.047**
**Number of chronic physical diseases**		
None	Reference	
1-2	2.8 (1.7-4.5)	**<0.001**
3 and more	11.3 (6.8-18.5)	**<0.001**
**12-month anxiety disorders**		
No	Reference	
Yes	8.5 (5.6-13.0)	**<0.001**
**12-month substance use disorders**		
No	Reference	
Yes	9.1 (4.2-19.5)	**<0.001**

OR=Odds Ratio; CI=Confidence Interval; a: All listed variables (gender, urbanicity, marital status, household income, number of chronic physical diseases, presence of 12-month anxiety disorders, and presence of 12-month substance use disorders) were simultaneously entered into the multivariable logistic regression model to obtain adjusted ORs; These analyses were based on part one weighted data.; b: Others included never married, separated, widowed, and divorced; c: The household income was categorized into three levels based on yearly income percentiles: low (<10,500 yuan), middle (10,500-42,500 yuan), and high (42,500 yuan and above).

Table e.5 Severity of role impairment with 12-month Depressive Disorders (N=293)

	N	Weighted proportion (%)	95% CI
**Household duties [a]**			
Very severe	17	4.4	1.3-7.5
Severe	27	17.0	9.8-24.2
Moderate	68	28.4	18.2-38.5
Mild	42	18.0	9.4-26.6
None	64	32.1	22.5-41.8
**Work [b]**			
Very severe	23	12.7	6.2-19.2
Severe	30	20.2	10.9-29.5
Moderate	46	25.3	12.7-37.8
Mild	37	15.0	7.1-23.0
None	51	26.8	16.5-37.1
**Relationships [c]**			
Very severe	9	3.3	0.0-7.3
Severe	24	18.1	7.7-28.4
Moderate	53	21.3	12.1-30.4
Mild	43	19.6	11.3-27.9
None	85	37.8	28.2-47.3
**Social life [d]**			
Very severe	8	1.5	0.0-3.2
Severe	27	19.1	10.7-27.6
Moderate	43	18.5	9.4-27.6
Mild	45	21.5	11.8-31.2
None	90	39.3	29.3-49.4

SE=Standard errors; a: Data missing from 75 respondents; b: Data missing from 106 respondents; c: Data missing from 79 respondents; d: Data missing from 80 respondents.

Table e.6 Severity of role impairment with 12-month Major Depressive Disorder (N=237)

	N	Weighted proportion (%)	SE	95% CI
**Household duties** [a]				
Very severe	17	5.1	-	1.7-8.6
Severe	26	19.6	4.0	11.2-28.0
Moderate	57	29.0	4.6	19.7-38.4
Mild	32	17.3	4.7	7.9-26.7
None	49	28.9	4.5	19.8-38.1
**Work** [b]				
Very severe	23	14.5	-	7.2-21.8
Severe	27	22.3	5.6	12.7-31.9
Moderate	41	26.2	7.1	11.7-40.7
Mild	28	14.5	4.6	5.3-23.8
None	38	22.5	4.9	12.6-32.4
**Relationships** [c]				
Very severe	9	3.9	-	2.0-5.7
Severe	23	21.1	5.9	9.1-33.1
Moderate	49	24.5	5.5	13.5-35.6
Mild	33	16.9	3.3	10.3-23.6
None	63	33.5	4.7	24.1-43.0
**Social life** [d]				
Very severe	8	1.8	-	0.0-3.8
Severe	24	22.3	5.0	12.3-32.2
Moderate	40	21.4	4.7	11.8-31.0
Mild	34	18.7	4.4	9.8-27.5
None	69	35.8	5.0	25.8-45.9

SE=Standard errors; a: Data missing from 56 respondents; b: Data missing from 80 respondents; c: Data missing from 60 respondents; d: Data missing from 62 respondents.

Table e.7 Severity of role impairment with 12-month Dysthymic Disorder (N=106)

	N	Weighted proportion (%)	95% CI
**Household duties** [a]			
Very severe	8	6.5	0.2-12.8
Severe	14	22.2	11.8-32.6
Moderate	29	35.3	19.6-51.0
Mild	9	11.7	0.0-25.8
None	20	24.3	7.5-41.1
**Work** [b]			
Very severe	13	20.1	11.3-28.9
Severe	13	23.8	15.9-31.8
Moderate	18	34.2	19.2-49.3
Mild	11	6.9	0.0-13.8
None	14	14.9	8.4-21.4
**Relationships** [c]			
Very severe	4	5.1	0.0-14.2
Severe	12	30.8	10.3-51.3
Moderate	23	15.9	9.6-22.2
Mild	17	21.7	6.1-37.3
None	24	26.5	11.2-41.9
**Social life** [d]			
Very severe	3	0.9	0.1-1.7
Severe	12	24.3	6.3-42.3
Moderate	19	24.7	6.7-42.7
Mild	18	21.5	5.9-37.1
None	27	28.6	12.9-44.3

SE=Standard errors; a: Data missing from 26 respondents; b: Data missing from 37 respondents; c: Data missing from 26 respondents; d: Data missing from 27 respondents.

Table e.8 Severity of role impairment with 12-month Depressive Disorder Not Otherwise Specified (N=48)

	N	Weighted proportion (%)	95% CI
**Household duties** [a]			
Very severe	0	-	-
Severe	1	0.4	0.0-1.5
Moderate	9	16.2	9.2-23.2
Mild	9	23.7	13.2-34.1
None	13	59.7	45.7-73.7
**Work** [b]			
Very severe	0	-	-
Severe	2	2.8	1.3-4.2
Moderate	4	7.6	0.0-15.5
Mild	7	20.9	18.2-23.5
None	12	68.8	60.1-77.5
**Relationships** [c]			
Very severe	0	-	-
Severe	1	1.4	0.0-5.1
Moderate	3	3.0	2.5-3.5
Mild	9	29.2	24.4-34.0
None	19	66.3	57.5-75.2
**Social life** [d]			
Very severe	0	-	-
Severe	2	1.8	0.0-5.6
Moderate	3	2.9	2.4-3.4
Mild	9	29.8	24.3-35.4
None	19	65.5	56.3-74.7

SE=Standard errors; a: Data missing from 16 respondents; b: Data missing from 23 respondents; c: Data missing from 16 respondents; d: Data missing from 15 respondents.
